# T Cell-Specific STAT1 Expression Promotes Lytic Replication and Supports the Establishment of Gammaherpesvirus Latent Reservoir in Splenic B Cells

**DOI:** 10.1128/mbio.02107-22

**Published:** 2022-08-15

**Authors:** P. A. Sylvester, C. N. Jondle, D. L. Schmalzriedt, B. N. Dittel, V. L. Tarakanova

**Affiliations:** a Department of Microbiology and Immunology, Medical College of Wisconsin, Milwaukee, Wisconsin, USA; b Versiti Blood Research Institute, Milwaukee, Wisconsin, USA; c Cancer Center, Medical College of Wisconsin, Milwaukee, Wisconsin, USA; Stony Brook University

**Keywords:** STAT1, T cell, acute infection, chronic infection, gammaherpesvirus, germinal center B cell

## Abstract

Gammaherpesviruses establish lifelong infections in most vertebrate species, including humans and rodents, and are associated with cancers, including B cell lymphomas. While type I and II interferon (IFN) systems of the host are critical for the control of acute and chronic gammaherpesvirus infection, the cell type-specific role(s) of IFN signaling during infection is poorly understood and is often masked by the profoundly altered viral pathogenesis in the hosts with global IFN deficiencies. STAT1 is a critical effector of all classical IFN responses along with its involvement in other cytokine signaling pathways. In this study, we defined the effect of T cell-specific STAT1 deficiency on the viral and host parameters of infection with murine gammaherpesvirus 68 (MHV68). MHV68 is a natural rodent pathogen that, similar to human gammaherpesviruses, manipulates and usurps B cell differentiation to establish a lifelong latent reservoir in B cells. Specifically, germinal center B cells host the majority of latent MHV68 reservoir in the lymphoid organs, particularly at the peak of viral latency. Unexpectedly, T cell-specific STAT1 expression, while limiting the overall expansion of the germinal center B cell population during chronic infection, rendered these B cells more effective at hosting the latent virus reservoir. Further, T cell-specific STAT1 expression in a wild type host limited circulating levels of IFNγ, with corresponding increases in lytic MHV68 replication and viral reactivation. Thus, our study unveils an unexpected proviral role of T cell-specific STAT1 expression during gammaherpesvirus infection of a natural intact host.

## INTRODUCTION

Gammaherpesviruses are ubiquitous pathogens that infect over 95% of the adult human population. These viruses cause several malignancies including B cell lymphomas and nasopharyngeal carcinomas (1). The two known human gammaherpesviruses, Epstein-Barr virus (EBV) and Kaposi’s Sarcoma associated herpesvirus (KSHV), replicate in several cell types and establish lifelong latency in select hematopoietic cells, including B cells and certain subsets of myeloid cells. Due to the high prevalence and exquisite species specificity of gammaherpesviruses, identifying the mechanisms that restrict or support chronic EBV or KSHV infection in the natural host proves difficult. To overcome this limitation, the current study uses murine gammaherpesvirus 68 (MHV68), a natural rodent pathogen that offers a powerful animal model of chronic gammaherpesvirus infection and pathogenesis. Importantly, there are many features shared between MHV68 and EBV/KSHV, including high level of genetic conservation (2–4). Further, both EBV and MHV68 infect monocytes, macrophages, and B cells and expand their latent reservoir in germinal center B cells which also serve as a target for B cell lymphomagenesis (5–15). Finally, MHV68-encoded viral proteins are either highly conserved or, even when not conserved, function to achieve the same biological goals as those encoded by EBV or KSHV (16, 17).

Interferons (IFNs) are a critical host antiviral defense (18–20) and comprise type I, II, and III IFNs. Type I IFN family consists of over a dozen different cytokines that signal through the ubiquitously expressed interferon α/β receptor (IFNAR). Type II IFN is represented by the single cytokine, IFN gamma, which signals via the interferon gamma receptor (IFNGR). While expression of type I and II IFN receptors is ubiquitous, expression of type III IFN receptors is limited to mucosal epithelial tissues and hepatocytes (21). Canonically, signaling initiated by all three types of IFNs assembles and requires STAT1-containing transcriptional complexes to induce expression of largely overlapping interferon stimulated genes (ISGs), some of which restrict viral replication (18, 22, 23). Given the antiviral role of IFNs, it is not surprising that type I and II IFNs attenuate EBV and KSHV lytic replication and reactivation *in vitro* (24–26), in spite of a plethora of gammaherpesvirus-encoded antagonists of the IFN network (27–29). Similarly, type I and II IFN signaling and STAT1 expression suppress lytic replication of MHV68 in primary macrophage cultures (30, 31). In contrast to type I and II, the role of type III IFN in gammaherpesvirus infection is far less clear.

While the role of IFNs in controlling EBV and KSHV chronic infection is presumed, it is challenging to ascertain in a natural host. Using the MHV68 model, however, it was found that global IFNAR1 expression attenuates acute MHV68 replication, promotes host survival, and suppresses persistent MHV68 replication and reactivation during long-term infection, particularly in the peritoneal cavity (32, 33). Similarly, lack of type II IFN signaling results in increased MHV68 persistent replication and viral reactivation during chronic infection (34, 35). While global IFN null mouse models have been instrumental in defining a critical role of IFN signaling in the control of gammaherpesvirus infection, the profoundly altered pathogenesis of MHV68 infection in these models precludes understanding of the role of cell type-specific IFN signaling during latent infection of an immunocompetent natural host. As such, we sought to target STAT1, a key transcription factor downstream of all IFN receptors, to define how STAT1-dependent mechanisms function in a cell type-selective manner to alter chronic gammaherpesvirus infection. Using this approach, we recently showed that macrophage-intrinsic STAT1 functions are antagonized by the conserved gammaherpesvirus protein kinase to promote MHV68 transition from macrophages to splenic B cells during the establishment of chronic infection (36).

Both CD4^+^ and CD8^+^ T cells are required for optimal control of chronic MHV68 infection. CD8^+^ T cells facilitate clearance of acute MHV68 replication in the lung (37, 38) and suppress reactivation in the peritoneal cavity and persistent replication (39). CD4^+^ T cell depletion during long-term infection leads to persistent MHV68 replication in the lungs due to the rise in dysfunctional, suppressive CD8^+^ T cell population (40–42). Classically, type I IFN acts as a “signal 3” alongside T cell receptor stimulation and co-stimulatory molecules to drive CD8^+^ T cell effector function and expansion (43–45). Consistent with this well-established role of type I IFN in other systems, CD8^+^ T cells of MHV68-infected IFNAR1^−/−^ mice displayed decreased expression of several cytokines, including IFN gamma and IL-2, with attenuation reversed by pharmacological suppression of lytic MHV68 replication (46). Intriguingly, T cell-specific IFNAR1 deficiency (dLck-Cre IFNAR1^flox/flox^ mouse model) did not recapitulate CD8 T cell defects observed in mice with global IFNAR1 deficiency (46), suggesting that either T cell-intrinsic type I IFN signaling plays no role in T cell responses during MHV68 infection or, given decreased T cell-mediated expression of IFN gamma in infected IFNAR1^−/−^ mice, the redundancy of type I and type II IFN signaling in facilitating CD8 T cell responses.

In this study we utilized a mouse model of conditional STAT1 deficiency that disrupts all STAT1 dependent functions, including classical IFN responses, in a cell type-dependent manner. The conditional STAT1 allele was used in conjunction with a distal Lck promoter driven Cre to achieve selective STAT1 deficiency in T cells (47). Using this model, we found that T cell intrinsic STAT1 expression restricts MHV68 driven B cell differentiation. However, despite an increased germinal center B cell response in infected mice with T cell-specific STAT1 deficiency, these germinal center B cells were not effective at supporting the MHV68 latency. Quite unexpectedly, we also found that T-cell intrinsic STAT1 expression restricts systemic IFN gamma production during MHV68 infection, with corresponding effects on viral control in the peritoneum and persistent replication in the lungs of chronically infected mice. Thus, our study reveals a bifurcated proviral role of T cell-intrinsic STAT1 during MHV68 infection, where it serves to facilitate infection of germinal center B cells yet restrict systemic IFN gamma production. These findings, in conjunction with our studies of myeloid cell-specific STAT1 deficiency (36) reveal multifaceted, cell type-dependent role of STAT1 during chronic gammaherpesvirus infection.

## RESULTS

### T cell-specific STAT1 deficiency leads to decreased acute and persistent MHV68 replication in the lungs.

**FIG 1:**
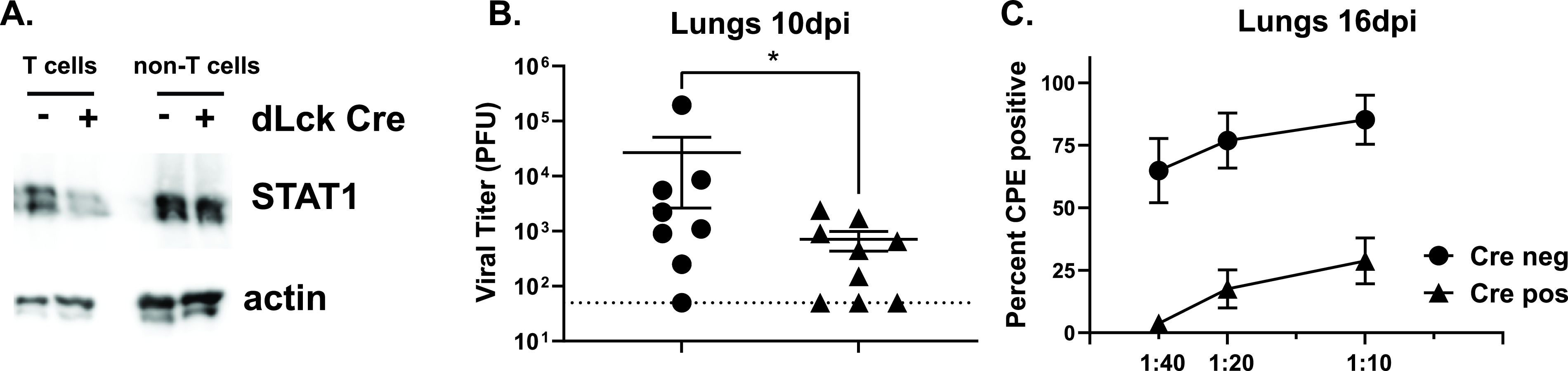
T cell-specific STAT1 deficiency leads to decreased acute and persistent MHV68 replication in the lungs. (A) Splenocytes were harvested from Cre negative and Cre positive mice. Total STAT1 levels in sorted T and non-T cells in the spleen were measured via Western blot. (B, C) Cre negative and positive littermates were infected intranasally with 1000 PFU of WT MHV68. (B) Viral lytic titers in lungs at 10 days postinfection, dotted line represents limit of detection, each symbol represents an individual animal. *, *P* < 0.05. (C) Persistent MHV68 replication in the lungs at 16 days postinfection was determined by semi-quantitative dilution assay. Data were pooled from 9 mice/group.

We observed a decrease in the levels of total STAT1 in T cells sorted from spleens of distal Lck promoter-driven Cre recombinase expressing STAT1^fl/fL^ mice (referred to as Cre positive) compared to Cre-negative STAT1^fl/fL^ littermates (referred to as Cre negative) (Fig. 1A). In contrast, STAT1 protein levels were similar in non-T cell splenic population from Cre positive and Cre negative mice, validating the experimental animal model. Further, baseline levels of total T cells and CD8^+^ T cell population in naive mice were not affected by T cell-specific STAT1 deficiency (see subsequent studies in Figs. 3 and 4).

Given a dramatic increase in acute and persistent MHV68 lung replication in IFNAR1^−/−^ mice (32, 33) along with attenuated expression of antiviral IFN gamma by IFNAR1^−/−^ CD8 T cells (46), we predicted that MHV68 replication would be elevated in the lungs of mice with T cell-specific STAT1 deficiency. In contrast to this prediction, acute MHV68 lung titers were decreased in Cre positive compared to Cre negative littermates at 10 days postinfection (Fig. 1B). While the majority of lytic MHV68 is cleared from the lungs by 12 days postinfection, low levels of persistent MHV68 replication in the lungs can be detected throughout chronic infection using highly sensitive mouse embryonic fibroblast (MEF)-based assay. Similar to that observed for acute lung titers, the low levels of persistent MHV68 replication in the lungs of chronically infected Cre negative mice were further reduced in Cre positive littermates at 16 days postinfection (Fig. 1C). Thus, T cell-specific STAT1 deficiency led to improved control of acute and persistent MHV68 replication in the lungs.

### T cell-specific STAT1 expression affects the latent reservoir and MHV68 reactivation in an anatomic site-specific manner.

**FIG 2:**
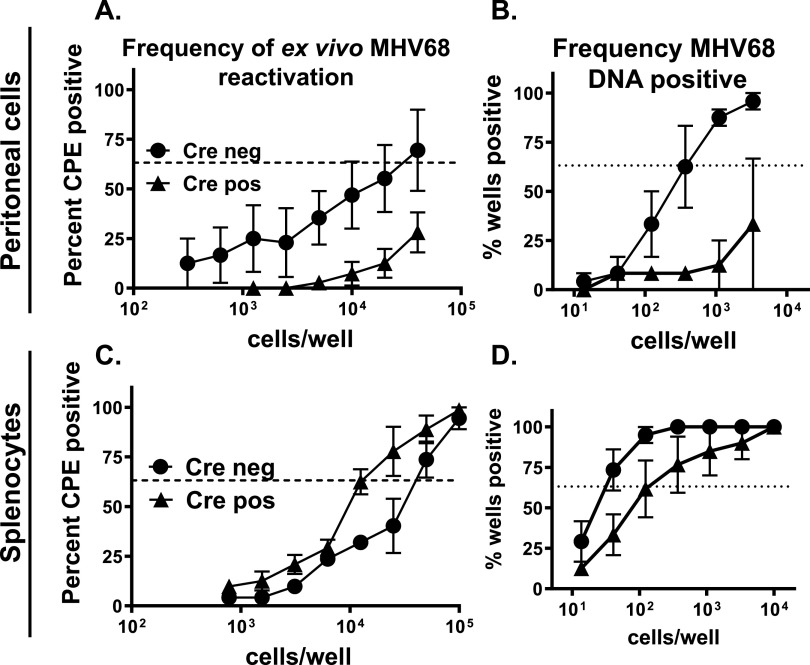
T cell-specific STAT1 expression affects the latent reservoir and MHV68 reactivation in an anatomic site-specific manner. Cre negative and positive littermates were infected as in Fig. 1. Peritoneal cells (A, B) or splenocytes (C, D) were harvested at 16 days postinfection and pooled from 3–5 mice within each group. (A, C) Intact pooled cellular populations were subjected to limiting dilution assay to determine the frequencies of *ex vivo* reactivation. (B, D) Frequencies of MHV68 DNA positive cells in each pooled population were determined by limiting dilution PCR. Data were pooled from 4–5 independent experiments. In the limiting dilution assays of this the dotted line is drawn at 63.2% and the x-coordinate of intersection of this line with the sigmoid graph represents an inverse of frequency of positive events.

The spleen and peritoneal cavity are the major sites of MHV68 latency during chronic infection, with the peak of the latent reservoir occurring around 16 days postinfection (5, 42, 48). While CD8 T cells have minimal effect on the MHV68 reactivation from splenocytes, MHV68 reactivation from the peritoneal cells is significantly elevated in the absence of CD8 T cells, highlighting the anatomic site-dependent control of chronic MHV68 infection (34). Similarly, IFN gamma is critical to control acute MHV68 replication in the lungs and viral reactivation from the peritoneal cells during chronic infection (35). Having observed improved control of lytic MHV68 replication in the lungs of Cre positive mice, MHV68 reactivation was measured in peritoneal cells of Cre negative and Cre positive littermates at 16 days postinfection. Consistent with the decrease in acute lung MHV68 titers (Fig. 1), frequency of MHV68 reactivation was decreased in Cre positive peritoneal cells (Fig. 2A), along with a significant decrease in the latent viral reservoir in the peritoneal cavity (Fig. 2B). Interestingly, while the frequency of MHV68 reactivation was elevated in the splenocytes of Cre positive mice (Fig. 2C), the overall latent MHV68 reservoir in the spleen was decreased (Fig. 2D). Persistent replication was not detected in either splenocytes or peritoneal cells of all experimental groups (data not shown). Thus, while T cell-specific STAT1 deficiency resulted in decreased MHV68 latent reservoir in both spleen and the peritoneal cavity, it led to opposite effects on MHV68 reactivation, with decrease in the peritoneal cavity, and increase in the spleen.

### T cell-specific STAT1 deficiency leads to increased systemic IFN gamma production and polyclonal CD8 T cell activation in the spleen.

**FIG 3:**
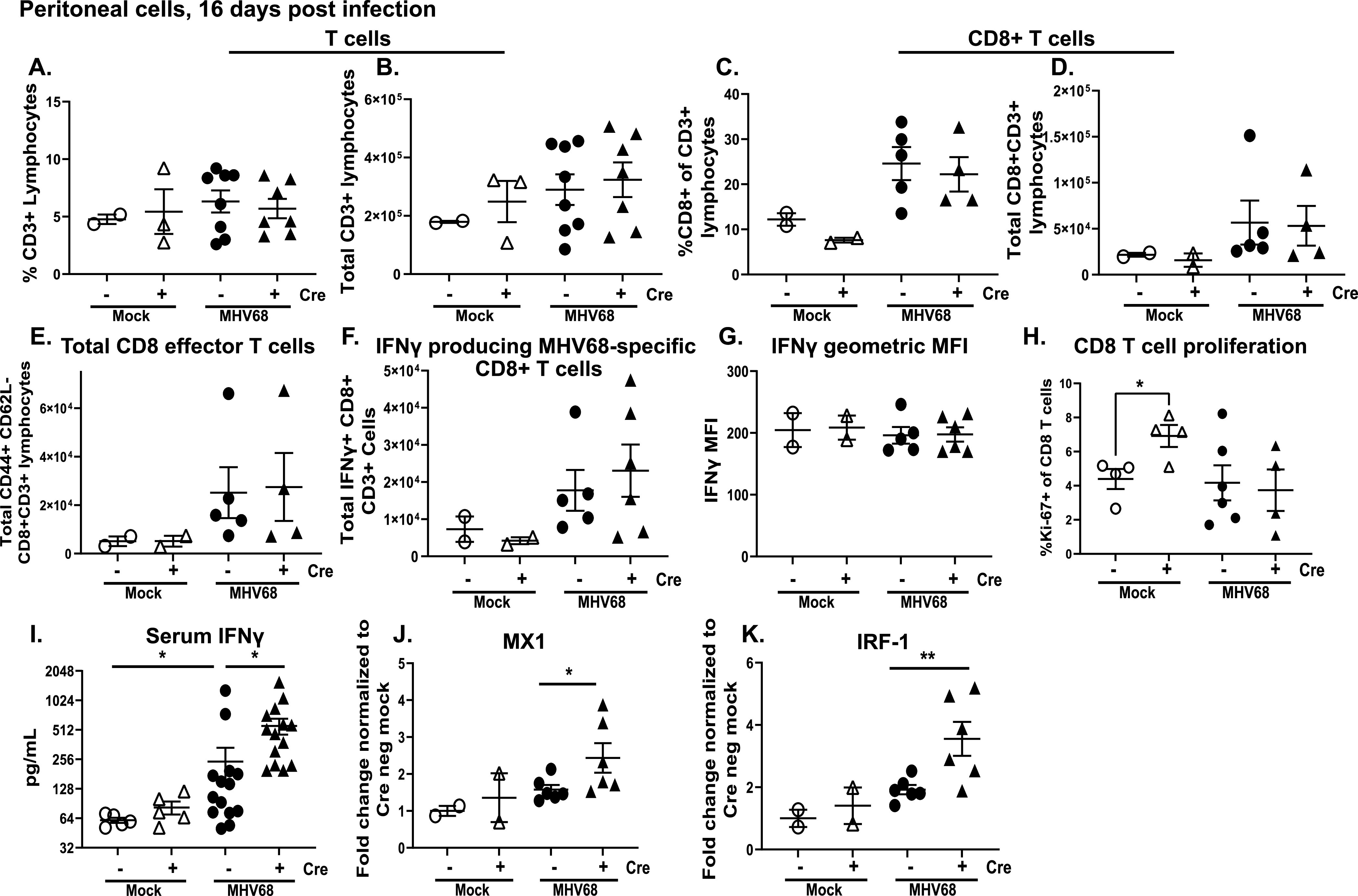
T cell-specific STAT1 deficiency leads to increased systemic gamma production. Cre negative and positive littermates were infected as in Fig. 1. Peritoneal cells were harvested at 16 days postinfection or mock treatment and subjected to flow cytometry to determine the frequencies (A, C, H) and absolute numbers (B, D, E, F) of total CD3^+^ T cells (A, B), CD8^+^ T cells (C, D), effector CD8 T cells (E), IFN gamma producing T cells (F), and proliferating CD8 T cells (H). (G) Flow cytometry was used to determine the mean fluorescence intensity of IFN gamma expression. (I) IFN gamma serum concentrations were determined by ELISA. (J, K) Gene expression of MX1 (J) and IRF-1 (K) from total peritoneal cells was determined by qRT-PCR. Each symbol represents an individual animal. *, *P* < 0.05.

IFN gamma producing T cells are important for controlling chronic MHV68 infection in the peritoneal cavity (39). Having observed a decrease in viral reactivation and latent reservoir in the peritoneal cavity (Fig. 2A and B), peritoneal T cell populations were assessed at 16 days postinfection. Despite the attenuated latent reservoir and reactivation in Cre positive peritoneal cells, the magnitude of the overall and CD8^+^ peritoneal T cell populations were comparable in the MHV68-infected Cre positive and Cre negative littermates (Fig. 3A to D). Next, the activation of CD8 T cells and abundance of MHV68-specific IFN gamma producing CD8^+^ T cells was assessed in the peritoneal cavity, the latter achieved by *ex vivo* restimulation with MHC-I MHV68 immunodominant peptides. Similar to that observed for the overall peritoneal T cell population, the abundance of activated effector CD8 T cells (CD44^+^CD62L-) or MHV68-specific CD8^+^ IFNγ^+^ T cells was not altered in Cre positive mice (Fig. 3E and F), with similar levels of IFN gamma expressed by peritoneal CD8 T cells on a per cell basis across all experimental groups (using mean fluorescent intensity of the IFN gamma signal, Fig. 3G). Further, while the proportion of proliferating (*Ki*-67+) peritoneal CD 8 T cells was higher in naive Cre positive mice, the low levels of proliferating CD8 T cells in the peritoneum were Cre genotype-independent in infected groups (Fig. 3H).

Interestingly, despite no difference in per-cell expression of IFN gamma in peritoneal CD8 T cells, IFN gamma levels were significantly higher in the sera of MHV68-infected Cre positive mice (Fig. 3I). To determine the effect to which increased systemic IFN gamma levels affect IFN signaling in peritoneal cells, mRNA levels of MX1 and IRF-1, well-established ISGs, were measured. We observed a modest increase in the mRNA level of MX1, a predominantly type I IFN-induced gene, in peritoneal cells of Cre positive chronically infected mice (Fig. 3J). Importantly, expression of IRF-1, a gene which is more sensitive to IFN gamma as compared to type I IFN signaling (49), was significantly increased in Cre positive peritoneal cells (Fig. 3K). Thus, T cell-specific STAT1 deficiency led to increased systemic IFN gamma levels and activity in the peritoneal cavity without affecting overall CD8 T cell activation and IFN gamma production by MHV68-specific CD8 T cells in the peritoneal cavity.

**FIG 4:**
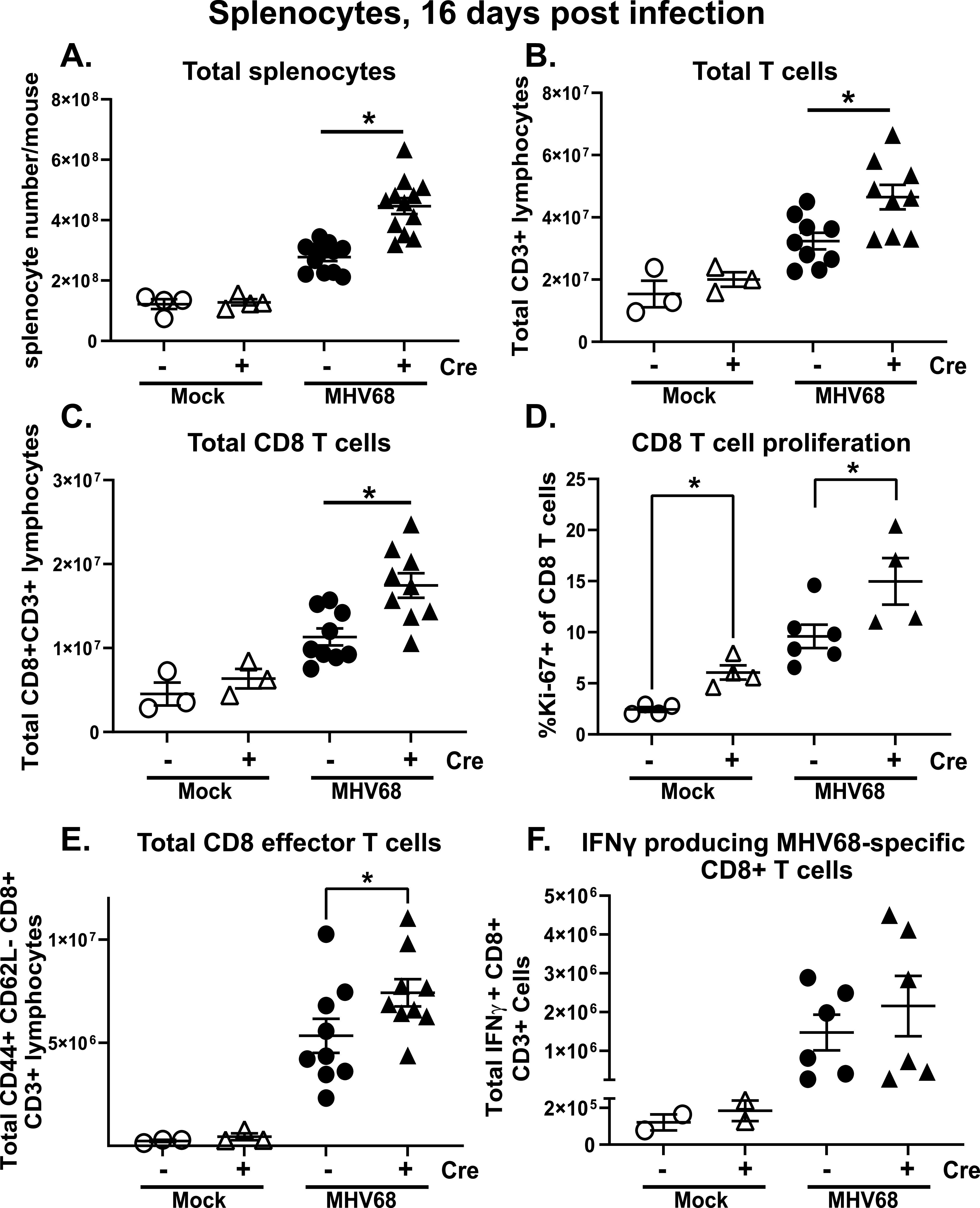
T cell-specific STAT1 deficiency leads to polyclonal CD8 T cell activation in the spleen. Cre negative and positive littermates were infected as in Fig. 1. Splenocytes were harvested at 16 days postinfection or mock treatment and subjected to flow cytometry to determine the frequencies (D) and absolute numbers (A-C, E, F) of total splenocytes (A), T cells (B), CD8^+^ T cells (C), proliferating CD8 T cells (D), effector CD8 T cells (E), and MHV68-specific IFN gamma producing CD8 T cells (F). Each symbol represents an individual animal. *, *P* < 0.05.

Antiviral T cell responses originate in the secondary lymphoid organs, including spleen, with subsequent recruitment of antiviral CD8 T cells to the distant sites of infection, such as peritoneal cavity. A defining and unique feature of gammaherpesvirus infection, including MHV68, is the robust polyclonal activation of T and B cells in secondary lymphoid organs, with many of these cells not specific for the gammaherpesvirus antigens (50, 51). Thus, given distinct nature of T cell responses in the lymphoid organs and infected distal tissues, we next examined T cell responses in the spleens of naive and MHV68-infected Cre positive and negative littermates. Interestingly, Cre positive littermates demonstrated increased splenomegaly as compared to Cre negative control mice upon MHV68 infection, but not at baseline (Fig. 4A). Consistent with increased splenomegaly, the total number of all T cells and the CD8 T cell subset was increased at 16 days postinfection in Cre positive mice (Fig. 4B and C). Further, the proportion of proliferating splenic CD8 T cells was increased both at baseline and following MHV68 infection in Cre positive mice (Fig. 4D). In contrast to that observed for peritoneal CD8 T cells (Fig. 3E), Cre positive MHV68-infected spleens displayed increased levels of effector CD8 T cells (CD44^+^CD62L-) (Fig. 4E). Interestingly, the abundance of MHV68-specific IFN gamma-producing CD8 T cells was not altered by the Cre genotype in infected animals (Fig. 4F). Thus, T cell-specific STAT1 deficiency led to a greater expansion of polyclonal, but not IFN gamma-producing, MHV68-specific CD8 T cells in the spleens.

### T cell-specific STAT1 deficiency leads to an increased germinal center B cell and plasma cell but not T follicular helper cell population during chronic MHV68 infection.

**FIG 5:**
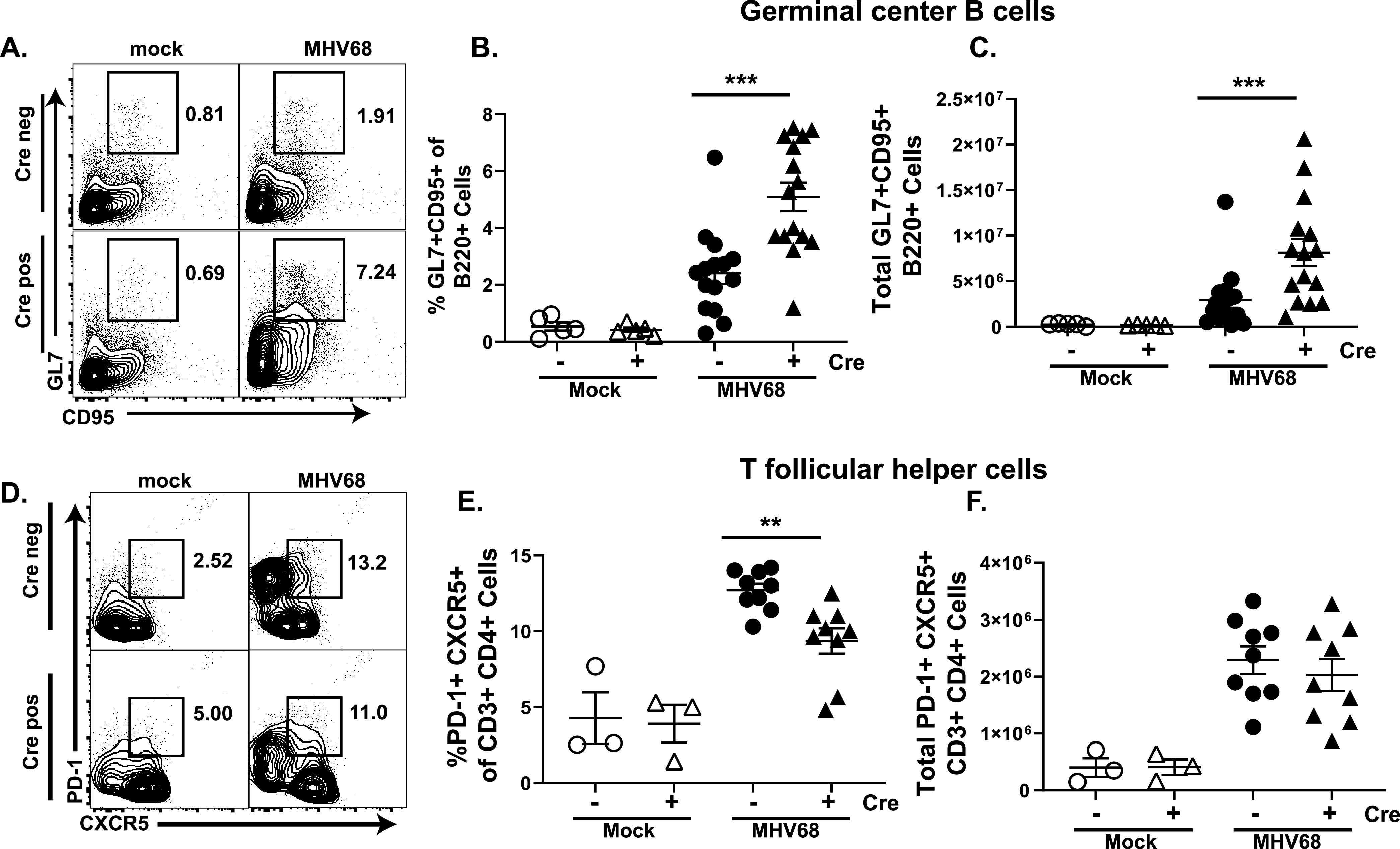
T cell-specific STAT1 deficiency leads to an increased germinal center B cell but not T follicular helper cell population during chronic MHV68 infection. Cre negative and positive littermates were infected as in Fig. 1. Splenocytes were harvested at 16 days postinfection or mock treatment and flow cytometry used to determine the frequencies (A, B, D, E) and absolute numbers (C, F) of germinal center B cells defined as B220^+^ GL7^+^ CD95^+^ (A–C) and T follicular helper cells defined as CD3^+^ CD4^+^ PD1^+^ CXCR5^+^ (D–F).

Increased systemic levels of IFN gamma (Fig. 3I) offered a plausible explanation for the decrease in lytic MHV68 replication in the lungs and attenuated chronic infection in the peritoneal cavity. In contrast, IFN gamma does not play a role in controlling MHV68 reactivation from the splenocytes (52), which was found to be increased in Cre positive mice (Fig. 2C). Instead, the establishment of MHV68 latency in the spleen and subsequent reactivation is intimately tied to viral manipulation of B cell differentiation. Specifically, MHV68 infects naive B cells and promotes entry of infected and bystander cells into the germinal center reaction, where the viral latent reservoir is exponentially amplified via germinal center B cell proliferation (48, 53). Further differentiation of latently infected B cells into plasma cells triggers reactivation, with plasma cells primarily responsible for overall MHV68 reactivation observed in the spleens of wild-type mice (53). Having observed an increase in viral reactivation and decreased latent reservoir in the spleen (Fig. 2C), we wanted to investigate if the T cell-specific loss of STAT1 affects MHV68-driven B cell differentiation. At 16 days postinfection, the germinal center B cell population was increased in the spleens of Cre positive as compared to Cre negative mice, both in frequency and absolute number (Fig. 5A to C). The increased abundance of germinal center B cells was surprising, as the MHV68 latent reservoir was lower in total splenocytes of Cre positive as compared to Cre negative littermates (Fig. 2D), a finding inconsistent with the well-established role of germinal center B cells in supporting MHV68 latency. CD4^+^ T follicular helper cells localize to germinal centers and are critical for expansion of germinal center B cells, including during MHV68 chronic infection (54). Interestingly, despite the expected positive feedback between germinal center B cell and T follicular helper populations, the frequency of T follicular helper cells was decreased in Cre positive mice, with no significant change in absolute cell numbers between MHV68-infected Cre negative and positive groups (Fig. 5D to F).

**FIG 6:**
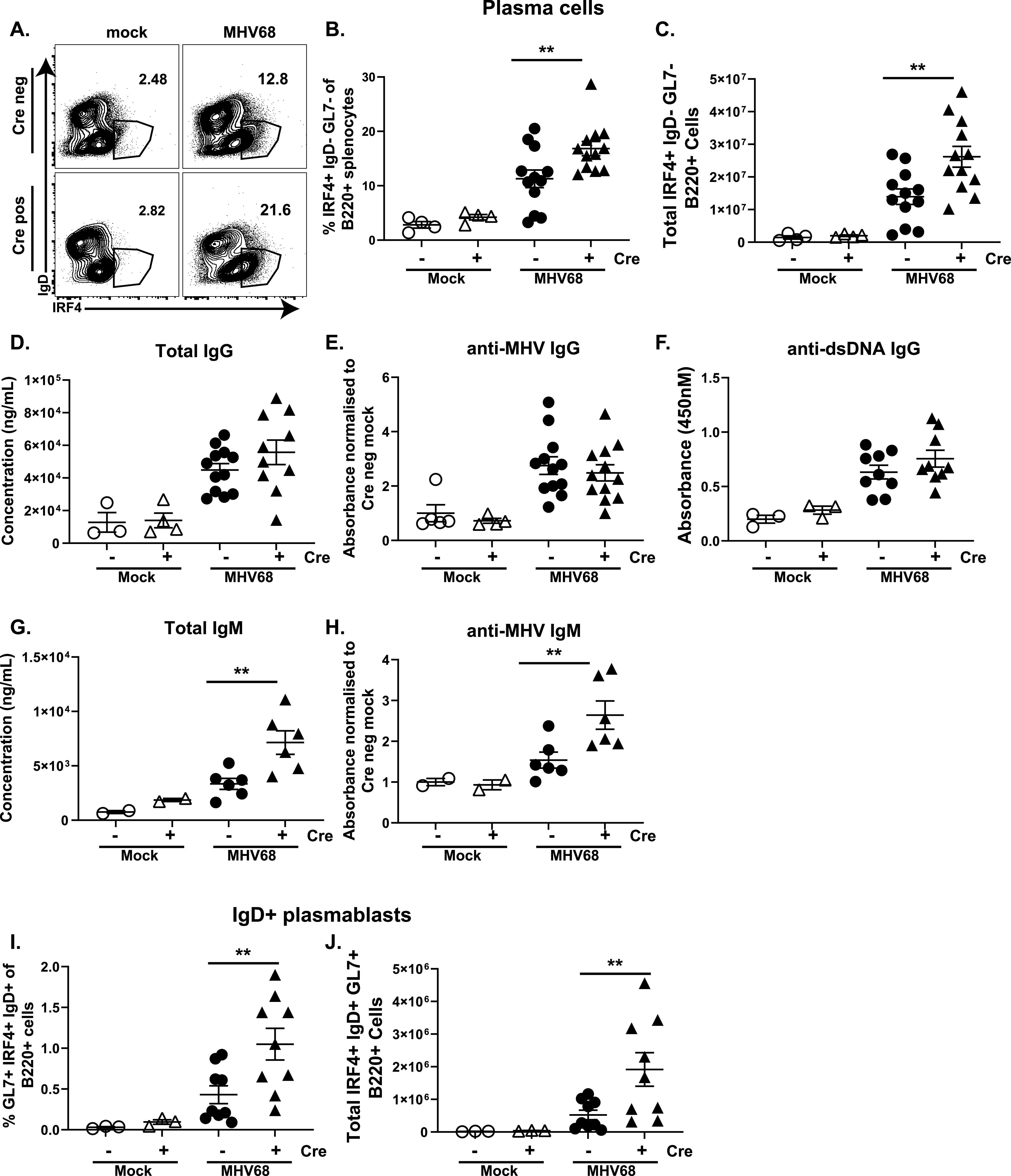
T cell-specific STAT1 deficiency leads to an increased abundance of terminally differentiated B cells. Cre negative and positive littermates were infected as in Fig. 1. Splenocytes were harvested at 16 days postinfection or mock treatment and flow cytometry used to determine the frequencies (A, B, I) and absolute numbers (D, J) of plasma cells defined as B220^+^ IRF4^+^ IgD- GL7- (A, B, C) and IgD^+^ plasmablasts defined as B220^+^ IgD^+^ IRF4^+^ GL7^+^ (I, J). Serum levels of total (D, G), virus-specific (E, H), IgG (D, E), and IgM (G, H) and anti-double stranded DNA IgG (F) were determined by ELISA. Each symbol represents an individual animal. **, *P* < 0.01.

Germinal center B cells differentiate into antibody secreting plasma cells that support MHV68 reactivation (53) and production of virus-specific and self- and foreign species-directed antibodies, with the latter a hallmark of chronic gammaherpesvirus infection (55). To assess the entire spectrum of B cell differentiation, plasma cell population and humoral responses were measured in Cre negative and positive littermates. Similar to that observed for germinal center B cells, both the frequencies (Fig. 6A and B) and absolute number (Fig. 6C) of class-switched (IgD^neg^) plasma cells were increased in Cre positive mice. Interestingly, despite increase in the abundance of class-switched plasma cells, titers of total, MHV68-specific, and double stranded DNA-specific class-switched IgG antibodies were similar in Cre negative and positive mice following MHV68 infection (Fig. 6D to F). Unexpectedly, titers of total and MHV68-specific IgM were increased in Cre positive mice (Fig. 6G and H). This correlated with increased abundance of Cre positive IgD^+^GL7^+^IRF-4^+^ plasmablasts that represent one plausible source of increased circulating IgM (Fig. 6I and J). In summary, T cell-specific STAT1 deficiency led to increased B cell differentiation without a concomitant increase in the T follicular helper cell population.

### T cell-specific STAT1 deficiency leads to decreased infection of B cells in the spleen.

**FIG 7:**
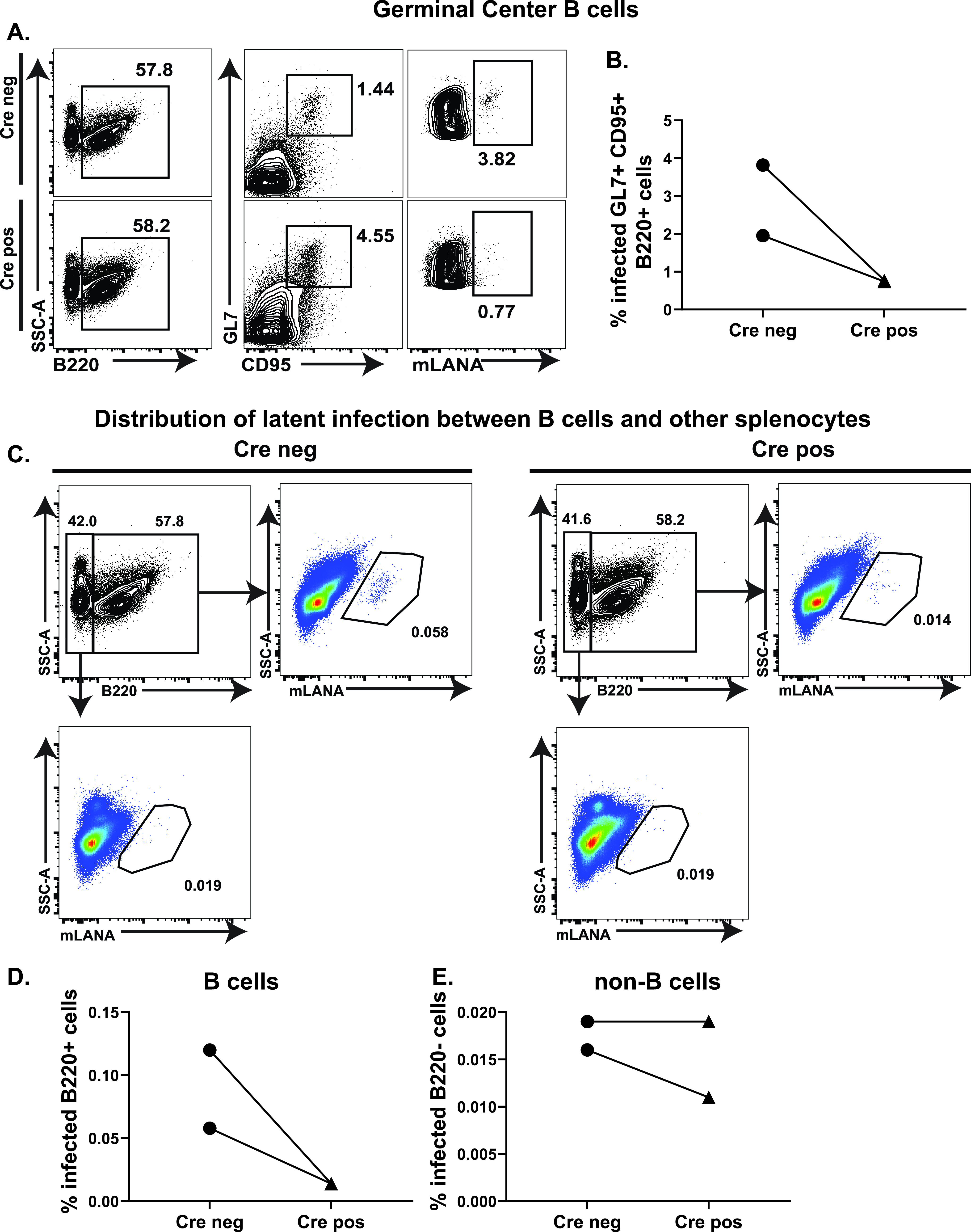
T cell-specific STAT1 deficiency alters MHV68 cell tropism in the spleen. Cre negative and positive littermates were infected intranasally with 1000 PFU of MHV68.orf73βla. At 16 days postinfection, splenocytes were harvested and pooled within each experimental group. The cells were then stained with cell surface markers and loaded with the fluorescent β-lactamase substrate CCF2-AM. Flow cytometry was used to determine mLANA expression as a result of the fluorescent signal from cleaved CCF2 in (A, B) germinal center B cells defined as B220^+^ GL7^+^ CD95^+^, (C, D) B220^+^ B cells, or (C, F) B220-non-B cells. Data in A and C are representative of two independent experiments.

Despite an increase in germinal center B cells in Cre positive mice (Fig. 5A), the latent reservoir of MHV68 in the spleen was decreased compared to Cre negative mice. This observed discrepancy from the expected outcome suggested that MHV68 tropism may be altered in Cre positive mice. To define infected splenocyte populations, Cre negative and positive littermates were infected with an MHV68 marker virus that expresses β-lactamase fused to MHV68 mLANA, a latent viral protein that is critical for viral episome maintenance during latency (56). Measurement of β-lactamase activity using a cell-permeable substrate offers a highly sensitive and accurate approach to define MHV68 tropism by flow cytometry (57–59). At 16 days postinfection, the frequency of β-lactamase+ latently infected germinal center B cells was significantly decreased in Cre positive, compared to Cre negative mice (Fig. 7A and B). Similarly, the infection of total splenic B220+ B cells was decreased in Cre positive mice (Fig. 7C and D). In contrast, the proportion of latently infected non-B cell splenocytes was not altered by the loss of T cell-specific STAT1 expression (Fig. 7E). Thus, despite the selective genetic targeting of T cells, a cell type that is not infected by MHV68, T cell-specific STAT1 deficiency resulted in decreased ability of splenic germinal center B cells to support viral latent reservoir.

## DISCUSSION

Our study reveals an unexpected proviral role of T cell-intrinsic STAT1 expression during gammaherpesvirus infection. We found that, while T cell intrinsic STAT1 deficiency resulted in expanded population of germinal center B cells, these B cells had lost the ability to efficiently maintain MHV68 latency. We also show that T cell-intrinsic STAT1 expression limits circulating levels of IFNγ, a major antiviral cytokine with a critical role in controlling chronic MHV68 infection. In addition to being a critical component of all classical IFN responses, STAT1 is also involved in other cytokine signaling pathways. Thus, while our study defines the role of T cell-intrinsic STAT1 during gammaherpesvirus infection, the involvement of STAT1-dependent IFN-independent pathways in the observed phenotypes cannot be ruled out.

### Effect of T cell-intrinsic STAT1 deficiency on T cell populations during MHV68 infection.

Global IFNAR deficiency leads to decreased expression of IFN gamma and IL-2 by CD8^+^ T cells during MHV68 infection (46). Unexpectedly, we observed that T cell-specific loss of STAT1 expression led to increased circulating IFN gamma levels and attenuated lytic MHV68 replication in the lungs and chronic infection in the peritoneal cavity. We subsequently hypothesized that STAT1 deficient CD8 T cells that represent an important source of IFN gamma during chronic MHV68 infection, are expressing higher levels of IFN gamma due to the lack of “sensing” of IFN levels and subsequent absence of a negative feedback loop. Surprisingly, this was not the case, as the abundance of peritoneal MHV68-specific CD8^+^ or CD4^+^ T cells and their ability to produce IFN gamma was not affected by the loss of STAT1 (Fig. 3, data not shown). Thus, in the context of MHV68 infection, T cell-intrinsic STAT1 expression is likely to act in *trans*, by suppressing the expansion and/or IFN gamma expression by other immune populations, such as NK cells and dendritic cells.

Surprisingly, we found that, while the abundance of activated effector CD8 T cells was not altered by Cre genotype in the peritoneal cavity, splenocytes of Cre positive MHV68-infected mice had an increased population of effector CD8 T cells (Fig. 4E), along with increased proliferation of CD8 T cells in Cre positive spleens (Fig. 4D). In contrast, the abundance of MHV68-specific IFN gamma producing CD8 T cells was similar in Cre positive and negative spleens (Fig. 4F), suggesting that T cell-intrinsic STAT1 expression selectively limits expansion of polyclonal, but not MHV68-specific CD8 T cells during chronic infection.

In the current study, we also observed differential effects of T cell-specific STAT1 deficiency on CD8^+^ vs CD4^+^ T follicular helper cells. While the antiviral CD8^+^ and CD4^+^ T cell subsets we analyzed were not affected (Fig. 3 and 4, and data not shown), the proportion of CD4^+^ T follicular helper cell population was decreased in Cre positive mice Fig. 5D, E. This result was unexpected given the increased population of germinal center B cells and the known positive feedback between these B cells and T follicular helper cells. T cell intrinsic type I IFN signaling has been shown to drive CD4^+^ T follicular helper differentiation in vaccination models (60), along with a similar function shown for STAT1 (61). In contrast, CD8^+^ T cells downregulate STAT1 during LCMV infection, leaving them less susceptible to the effects of IFN signaling (62). Thus, in our model, it is possible that T cell-intrinsic STAT1-dependent IFN signaling also drives T follicular helper proliferation during MHV68 infection. However, as CD8^+^ T cells naturally downregulate STAT1 during infection, they may become resistant to STAT1 dependent IFN signaling, leading to lack of STAT1-dependent phenotypes in antiviral CD8^+^ T cells, at least in our assays.

### Effect of T cell-intrinsic STAT1 deficiency on B cell populations during MHV68 infection.

T cell-specific STAT1 deficiency in the context of chronic MHV68 infection has revealed an unexpected cross talk with virus-driven B cell differentiation. Despite observing a decrease in T follicular helper cells, the abundance of germinal center B cells was increased in MHV68-infected Cre positive mice. This discrepancy was not a result of decreased FoxP3^+^ CD4 T follicular regulatory cells as this population was comparable between Cre negative and Cre positive mice (data not shown). In addition, despite an overall increase in germinal center B cells, the proportion of infected germinal center B cells was decreased when T cells lacked STAT1 expression. This decreased “fitness” of germinal center B cells to maintain latent MHV68 infection was noted in several other mouse models of MHV68 infection, highlighting the fact that the mere presence of germinal center B cells does not make them an optimal host of viral latency. Interestingly, we previously showed that global IRF-3 deficiency results in decreased ability of germinal center B cells to support MHV68 latency, despite a numerically wild type germinal center response in IRF-3^−/−^ mice (57). Given the important role of IRF-3 in type I IFN expression, it is possible that decreased autocrine IFN signaling in T follicular helper cells of IRF-3^−/−^ mice led to suboptimal “help” provided to infected germinal center B cells. However, given the involvement of both STAT1 and IRF-3 in multiple signaling pathways, the role of T cell-intrinsic IFN signaling in supporting latent infection of germinal center B cells needs to be specifically defined. Several factors may modulate (i) whether MHV68 can infect and establish latency in a naive B cell and (ii) whether this infected B cell can undergo proper activation, differentiate into a germinal center B cell, and survive and expand in the germinal center. In our model of T cell-specific STAT1 deficiency, the decreased T follicular helper population may still be sufficient to support expansion of uninfected germinal center B cells, but MHV68 infected germinal center B cells may require quantitatively and qualitatively different T follicular helper functions in order to expand and maintain latent infection. A decrease in such T follicular helper functions, to be defined in the future, could, therefore, be responsible for the selective decrease in infected germinal center B cells in mice with T cell-specific STAT1 deficiency.

In our study we observed increased reactivation from splenocytes of Cre positive mice, despite a decreased infection of germinal center B cells. Gammaherpesvirus reactivation from B cells is triggered by transcription factors (IRF-4, XBP-1) that are selectively enriched in B cells that had terminally differentiated into an antibody-producing cell (63, 64). We observed an increase in both class-switched plasma cells and IgD+ plasmablast populations in Cre positive mice (Fig. 6). Given the lower efficiency of germinal center B cell infection, the germinal center-derived class-switched plasma cells are unlikely to contribute to increased MHV68 reactivation observed in Cre positive splenocytes. However, increased reactivation could result from increased MHV68 infection of B cells that differentiate via an extrafollicular route, including IgD+ plasmablasts that are increased in mice with T cell-specific STAT1 deficiency (Fig. 6I and J). As extrafollicular differentiation is skewed away from class-switching, these IgD+ plasmablasts are also likely to be the source of the increased IgM antibody titers seen in Cre positive mice. However, the extrafollicular plasma cells are typically short-lived compared to germinal center derived plasma cells (65, 66) and may not support the re-seeding of the latent MHV68 during long-term infection. It is also possible that the increased frequency of reactivation observed in Cre positive splenocytes is due to increased reactivation from non-B cells, such as dendritic cells and macrophages that MHV68 also latently infects (67). However, this latter possibility is less likely given the similar frequency of infection in the non-B cell compartment and a greater susceptibility of these cell types to the antiviral effects of IFN gamma.

### Model.

**FIG 8:**
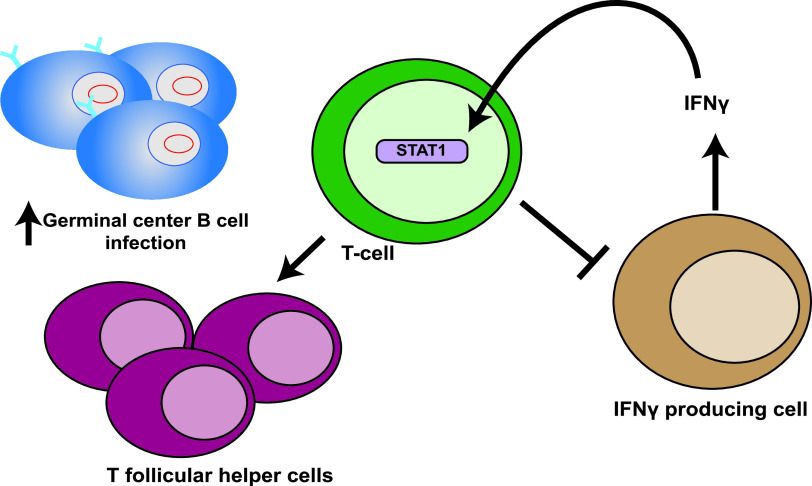
Working model of the role of T-cell intrinsic STAT1 expression during chronic MHV68 infection. T-cell intrinsic STAT1 expression serves two distinct functions during chronic MHV68 infection. First, T cell-intrinsic STAT1 is necessary for quantitative and qualitative features of CD4 T follicular helper population that support latently infected germinal center B cells. Second, STAT1 dependent IFNγ signaling is a negative-feedback loop whereby, in response to high systemic IFNγ levels, T cells suppress the production of IFNγ by other immune cells.

With these data, we would like to propose a theoretical model for the role of STAT1 in T cells during chronic MHV68 infection (Fig. 8). T cell-intrinsic STAT1 activation (by IFN and/or other cytokines) drives the differentiation of CD4^+^ T cells into T follicular helper cells, which are optimally suited to support latently infected germinal center B cells. On the other hand, T cell intrinsic STAT1 dependent IFNGR signaling is a component of a negative feedback loop. It is possible that T-cells use an increase in STAT1 levels as a sensor of circulating IFNγ, and once IFNγ/STAT1 levels reach a certain threshold, antagonize the production of this cytokine by other immune cells. This negative feedback loop is usurped by MHV68 to increase reactivation from peritoneal cells and persistent replication in lungs during chronic infection. Although this negative feedback loop may seem counterproductive in the context of controlling infection, it may serve to protect the host from interferonopathies associated with excess IFN gamma production over a lifelong infection.

In summary, our study revealed an unexpectedly proviral role of T cell-intrinsic STAT1 during gammaherpesvirus infection. Future studies will define the relative contribution of IFN-dependent versus non-IFN STAT1 signaling to the host and viral phenotypes defined in this study.

## MATERIALS AND METHODS

### Animals.

STAT1^fl/fL^ mice (on a mixed B10.PLxC57BL/6J genetic background) were originally obtained by Dr. Dittel. STAT1^fl/fL^ mice were bred to mice expressing Cre recombinase driven by the distal LcK promoter to generate distal LcK Cre STAT1^fl/fL^ mice (68). Given the mixed genetic background, dLck Cre positive and negative STAT1^fl/fL^ littermates were used in each experiment. Mice were housed in a specific-pathogen-free barrier facility in accordance with institutional and federal guidelines. All experimental manipulations of mice were approved by the Institutional Animal Care and Use Committee of the Medical College of Wisconsin (AUA971).

### Viruses and infections.

Virus stocks were prepared and titered on NIH 3T12 cells. Infections were performed by intranasal inoculation of 3–5 mice per group at 6–7 weeks of age under light anesthesia. Mice were inoculated with 1000 PFU of MHV68 (WUMS) or sterile carrier (mock) in an inoculum volume of 15 μL per mouse. Virus was diluted in sterile serum-free Dulbecco’s modified Eagle’s medium (Corning, Tewksbury, MA) immediately prior to infection. The virus expressing β-lactamase (MHV68.ORF73βla) (56) was a kind gift of Dr. Scott Tibbetts.

### Viral assays.

The frequency of MHV68-DNA positive cells and frequency of *ex vivo* reactivation of MHV68 were determined by limiting dilution assay. To determine the frequency of cells harboring viral DNA, splenocytes and peritoneal exudate cells were isolated and pooled from all mice within each experimental group (three to five mice/group) and six serial 3-fold dilutions were subjected to a two-round, nested PCR (12 replicates/dilution) using primers against the viral genome. Primers used for limiting dilution PCR were as follows:

Round 1/Outer Forward: 5′-GAGATCTGTACTCAGGCACCTGT-3′
Round 1/Outer Reverse: 5′-GGATTTCTTGACAGCTCCCTGT-3′
Round 2/Inner Forward: 5′-TGTCAGCTGTTGTTGCTCCT-3′
Round 2/Inner Reverse: 5′-CTCCGTCAGGATAACAACGTCT-3′

PCRs were then analyzed by agarose gel electrophoresis and the number of reactions positive for MHV68 DNA was scored for each dilution.

To determine the frequency of cells reactivating virus *ex vivo*, splenocytes or peritoneal exudate cells were pooled from all mice within each experimental group (three to five mice/group) and 8–12 serial 2-fold dilutions of cell suspensions from each group were plated onto monolayers of mouse embryonic fibroblasts (MEFs) at 24 replicates per dilution. To control for preformed virus, 2-fold serial dilutions of mechanically disrupted splenocytes or peritoneal exudate cells were plated as above. Cytopathic clearing of MEFs was scored at 21 days post-plating. The use of primary MEFs to amplify virus lowers sensitivity of lytic MHV68 detection below a single plaque forming unit. To determine the level of persistent replication in the lungs during chronic infection, lungs were isolated at 16 days postinfection, placed into sterile DMEM and homogenized. Homogenates were serially diluted up to 160-fold and plated onto monolayers of MEFs at 12 replicates per dilution. The cytopathic clearing of MEFs was scored at 21 days post-plating. For acute infection studies, organs were isolated at 7 days postinfection, placed into sterile DMEM and homogenized. Homogenates were serially diluted 10-fold and viral titer was determined via plaque assay using NIH 3T12 fibroblasts.

### Flow cytometry.

Single Cell suspensions of splenocytes were prepared in fluorescence-activated cell sorting (FACS) buffer (PBS, 2% fetal bovine serum, 0.05% sodium azide) at 1 × 10^7^ nucleated cells/mL. A total of 1.5 × 10^6^ cells were pre-stained with Fc block (24G2 antibody), then incubated with an optimal dilution of antibody on ice. The following antibodies and reagents were used in this study and purchased from BioLegend (San Diego, CA): B220-PE-Cy7, CD95-PE, GL7-FITC, CD3-APC A700, CD4-PB, CXCR5-PE-Texas Red, PD-1-PE, CD44-PE-Cy7, CD62L- PE-Texas Red. Data acquisition was performed on an LSR II flow cytometer (BD Biosciences, San Jose, CA), and the data were analyzed using FlowJo software (Tree Star, Ashland, OR). In some cases, after extracellular staining, splenocytes from mice infected with the β-lactamase reporter virus were incubated with cell-permeable CCF-2 substrate for 1 h.

### Western blot.

Splenocytes were isolated from Cre negative and positive littermates and sorted into T and non-T cell populations using the MojoSort mouse CD3 T cell isolation kit (BioLegend, San Diego, CA). Cells were then resuspended in 2X Laemmli buffer (0.1M Tris, 4% SDS, 4 mM EDTA, 0.095M 2-mercaptoethanol, 1.07M glycerol, 0.017% Bromophenol blue, pH 6.8). Proteins in each sample were separated using 10% SDS-PAGE and transferred to a PVDF membrane using semi-dry method. Proteins of interest were detected by immunoblot analysis using antibodies against total STAT1 (1:10000, Santa Cruz Biotechnology) or β-actin (1:20000, Cell Signaling #4970).

### ELISA.

IFN gamma concentrations were determined using IFN gamma ELISA MAX Deluxe set (Biolegend, San Diego CA) according to the manufacturer’s instructions, using Nunc MaxiSorp flat-bottom plates (Thermo Fisher Scientific, Waltham, MA). Serum was diluted 1:2 in assay diluent prior to analysis. Horseradish peroxidase (HRP) enzymatic activity was stopped by the addition of 1 N HCl and absorbance was read at 450 nm on a model 1420 Victor 3V multilabel plate reader (PerkinElemer, Waltham, MA).

### qRT-PCR.

Total RNA was harvested using TRIzol extraction method (Invitrogen, Waltham, MA). Isolated RNA was DNase treated and reverse transcribed using M-MLV reverse transcriptase (Promega, Madison, WI), and analyzed by quantitative reverse transcriptional- PCR (qRT-PCR). cDNA was assessed in triplicate, along with corresponding negative RT reactions by real-time PCR using CFX Connect System (Bio-Rad, Hercules, CA). The relative abundance of each cDNA in the sample was normalized to the corresponding GAPDH levels using the delta CT (ΔC_T_) method. Primers used for qRT-PCR were as follows: Mx1 Forward: 5′-AGCTAGACAGAGCAAACCAAGCCA-3′; Mx1 Reverse: 5′-TCCCTGAAGCAGACACAGCTGAAA-3′; IRF-1 Forward: 5′-ACACTAAGAGCAAAACCAAGAG-3′; IRF-1 Reverse: 5′-TTTCCATATCCAAGTCCTGA-3′; GAPDH Forward: 5′-TGTGATGGGTGTGAACCACGAGAA-3′; GAPDH Reverse: 5′-GAGCCCTTCCACAATGCCAAAGTT-3′.

### Statistical analyses.

Statistical analyses were performed using unpaired one-tailed Student's *t* test (Prism, GraphPad Software, Inc.).
